# Supplementary material for: miR-9 regulates basal ganglia-dependent developmental vocal learning and adult vocal performance in songbirds
Source: eLife. 2018 Jan 18;7:e29087. doi: 10.7554/eLife.29087 (PMC5800847; doi:10.7554/eLife.29087)
Supplement: Transparent reporting form [file elife-29087-transrepform.pdf]

1st Floor
24 Hills Road
Cambridge CB2 1JP, UK

P 01223 855340
W elifesciences.org
T @elife

eLife's transparent reporting form

We encourage authors to provide detailed information *within their submission* to facilitate the interpretation and replication of experiments. If you have any questions, please contact us: editorial@elifesciences.org.

Sample-size estimation

- You should state whether an appropriate sample size was computed when the study was being designed
- You should state the statistical method of sample size computation and any required assumptions
- If no explicit power analysis was used, you should describe how you decided what sample (replicate) size (number) to use

Please outline where this information can be found within the submission (e.g., page numbers or figure legends), or explain why this information doesn't apply to your submission:

We did not perform power analysis to determine sample size. Our sample size was similar to those generally employed in the field. This is reported in the Statistical analysis/Methods section (p. 31). Sample sizes (number of animals or number of analyzed motif or syllables) for each experiment were reported in respective figure legends and in relevant places in the Results section.

Replicates

- You should report how often each experiment was performed
- You should include a definition of biological versus technical replication
- The data obtained should be provided and sufficient information should be provided to indicate the number of independent biological and/or technical replicates
- If you encountered any outliers, you should describe how these were handled
- Criteria for exclusion/inclusion of data should be clearly stated
- High-throughput sequence data should be uploaded before submission, with a private link for reviewers provided (these are available from both GEO and ArrayExpress)

Please outline where this information can be found within the submission (e.g., page numbers or figure legends), or explain why this information doesn't apply to your submission:

eLife Sciences Publications, Ltd is a limited liability non-profit non-stock corporation incorporated in the State of Delaware, USA, with company number 5030732, and is registered in the UK with company number FC030576 and branch number BR015634 at the address 1st Floor, 24 Hills Road, Cambridge CB2 1JP | August 2014

For data presented in Figure 2C/D, Figure 3A, and Figure 5B, two investigators (one blind to the treatment groups) analyzed behavioral data. This information is reported in statistical analysis/methods section (p. 31).

For song behavioral experiments, we had n = 6 animals per group. For data presented in Figure 2, for each animal, > 50 motif renditions were analyzed. For data presented in Figures 3A, 4D, and 6A, 20 pupil motif renditions and 10 tutor motif renditions were analyzed to obtain 200 pairwise comparisons. When analysis was performed at the syllable level (Figure 5 and Figure 6B-E), 20-40 syllable renditions were used, and both the number of syllables and the number of animals included in the analyses were reported in figure legends. Detailed description of song recording and analysis methods are provided in the Methods section (p. 22-27).

Outliers due to technical errors were removed. Otherwise, they remained in the data.

For gene expression analysis, the number of animals and replications were reported in the figure legends and the Methods section (p. 30).

We examined viral injection site at the end of song behavior experiments. Two animals (one control and one miR-9) were excluded from behavior analysis because the injection sites were outside of Area X. This was reported in the statistical analysis/methods section (p. 31).

Statistical reporting

- Statistical analysis methods should be described and justified
- Raw data should be presented in figures whenever informative to do so (typically when N per group is less than 10)
- For each experiment, you should identify the statistical tests used, exact values of N, definitions of center, methods of multiple test correction, and dispersion and precision measures (e.g., mean, median, SD, SEM, confidence intervals; and, for the major substantive results, a measure of effect size (e.g., Pearson's r, Cohen's d)
- Report exact p-values wherever possible alongside the summary statistics and 95% confidence intervals. These should be reported for all key questions and not only when the p-value is less than 0.05.

Please outline where this information can be found within the submission (e.g., page numbers or figure legends), or explain why this information doesn't apply to your submission:

Statistical tests used, N values (including the number of animals or the number of syllables) and p values for each experiment were described in the figure legends and in relevant places in the results section. Whenever possible, individual data points, averaged value, and SEM or SD were presented in the figures or explained in the legends. Statistical analysis also was summarized in the statistical analysis/methods section (p.31).

(For large datasets, or papers with a very large number of statistical tests, you may upload a single table file with tests, Ns, etc., with reference to page numbers in the manuscript.)

Additional data files ("source data")

- We encourage you to upload relevant additional data files, such as numerical data that are represented as a graph in a figure, or as a summary table
- Where provided, these should be in the most useful format, and they can be uploaded as "Source data" files linked to a main figure or table
- Include model definition files including the full list of parameters used
- Include code used for data analysis (e.g., R, MatLab)
- Avoid stating that data files are "available upon request"

Please indicate the figures or tables for which source data files have been provided:

Source data files have been provided for: Figure 2, Figure 3A, Figure 4D, and Figure 5.

eLife Sciences Publications, Ltd is a limited liability non-profit non-stock corporation incorporated in the State of Delaware, USA, with company number 5030732, and is registered in the UK with company number FC030576 and branch number BR015634 at the address 1st Floor, 24 Hills Road, Cambridge CB2 1JP | August 2014
